# Redefining assessment tasks to promote students’ creativity and integrity in the age of generative artificial intelligence

**DOI:** 10.1007/s40979-025-00201-x

**Published:** 2025-09-23

**Authors:** Martine Peters, Dimitar Angelov

**Affiliations:** 1 Department of Education, Université du Québec en Outaouais, Gatineau, Canada; 2 Coventry University, Coventry, UK

**Keywords:** Integrity, Assessment, Creativity, Generative Artificial Intelligence, Plagiarism

## Abstract

The arrival of generative artificial intelligence (GenAI) has forced lecturers to adjust their assessment practices to ensure that students’ work is their own from a creative point of view, and free of plagiarism. This chapter proposes the Academic Integrity and Creativity in the Age of Artificial Intelligence (AICAI) model for the use of authentic assessment as a possible strategy to promote students’ creativity and integrity and thereby ensure the ownership of their written work. Lecturers are encouraged to rethink the assignments they design and examine each of the following components with an eye to integrity: their professional characteristics, the objectives for the assignment, the type of assessment that is appropriate for the needs of the student. Others include the cognitive offloading that will be done or not with GenAI, the type of authentic task they wish to propose and its characteristics, and the instructions and criteria that will be given to students. The choices made should engage students, thereby diminishing the temptation to plagiarize. By combining different strands of pedagogical theory and research, the **AICAI** assessment design model proposed in this paper has brought into focus the challenges as well as the opportunities that have emerged with the inclusion of GenAI in higher education. On a more practical level, it offers a systemic approach and advice as to how the challenges can be mitigated and benefits maximized for all parties involved in assessment.

## Clinical trial number

Not applicable.

## Introduction

Assessment is a fundamental component of our education system, used daily in classrooms around the world. Assessment should be used as a tool to promote learning (Sotiriadou et al. 2020) – for example, to give students feedback on their performance – rather than only as a tool to gauge students’ achievement, deployed to give grades (Sato et al. 2006). In higher education, assessment has tended to be more traditional, e.g. exams, essays and presentations, with little change in the evaluation methods over the years (Williams 2014), which can be attributed to lecturers’^1^ lack of time (Bovill 2020) or training (Stewart 2014; Vilppu et al. 2019). In the last few years, two major upheavals in the education system, the pandemic and the arrival of generative artificial intelligence^2^ (GenAI), have forced lecturers to adjust their assessment practices to ensure that students’ work is their own, from a creative point of view, and free of plagiarism. This article will propose a theoretical model for the use of authentic assessment as a possible strategy to promote students’ creativity and integrity and thereby ensure the ownership of their written work.

## Literature review: changing the landscape of assessment: two significant upheavals in education

In the past hundred years, the education systems around the world have undergone a number of fundamental changes (Subba 2017); for example, the globalization and democratization of education, the right of women to attend schools, and the introduction of technology, etc. These transformations happened incrementally or were separated with long periods of time, contrary to a couple of recent upheavals – the Covid-19 pandemic and the arrival of GenAI – which have taken place in the last five years. Similarly to all previous turning points in the history of education, the pandemic and GenAI should leave long-lasting effects for the day-to-day practice of higher education globally, which are yet to be fully understood and taken into consideration.

### Pandemic: the first major upheaval in twenty-first-century higher education

The Covid-19 pandemic has had a major impact on education all over the world (Marinoni et al. 2020), with many negative consequences for lecturers and students (Vinichenko et al. 2021). By necessity, there was a shift to online learning and teaching (Pokhrel and Chhetri 2021), which provoked an increased use of technology (Okoye et al. 2021), such as video conferencing and learning platforms (Adipat 2021). Lecturers had to be more flexible and adaptable, as well as use different teaching and assessment strategies to engage students in their distance learning (Rinaldi 2022).

In higher education, one repercussion of the pandemic was the rise in plagiarism cases (Hill et al. 2021). Online learning, as well as the increased use of technology overall, encouraged fraudulent academic behaviors (Erguvan 2021). Students’ lack of engagement in their studies was another reason why plagiarism cases were on the rise during the pandemic (Muassomah et al. 2022. When confronted with suspected plagiarism, lecturers responded by using a variety of strategies, from proctoring software (Kamalov et al. 2021), reweighting assessments (or introducing new ones) to being very lenient (San Jose 2022).

### GenAI: the second major upheaval in twenty-first-century higher education

More recently, a second upheaval occurred in global higher education: the arrival of GenAI (Wang et al. 2024). According to numerous sources, the advent of GenAI will revolutionize learning and teaching at all levels (Bearman and Luckin 2020; Bearman et al. 2022; Farrelly and Baker 2023; Wang 2024), with GenAI literacy becoming an essential element for all students to master (Southworth et al. 2023).

In higher education, reactions to the introduction of GenAI have been varied. Many lecturers have panicked, demanding that their institution ban the use of GenAI on campus (Sullivan et al. 2023), while others have embraced this new technology, discussing it with students and demonstrating its usefulness (Rudolph et al. 2023). Yet others have started to deploy GenAI to grade papers (Celik et al. 2022), plan their lessons (Celik et al. 2022) or conceive assessment tasks (Cotton et al. 2024).

What has become clear in a very short time is that GenAI has had both negative and positive impacts on plagiarism in higher education. On the negative side, as soon as the accessibility of GenAI became known, students have taken recourse to it to write their assignments, submitting GenAI-generated text for assessments (Cotton et al. 2024). Such behavior constitutes a type of plagiarism that is practically undetectable at this point in time (Cingillioglu 2023). Having GenAI write an essay is similar to buying one from an essay mill, with both types of plagiarism very difficult to identify (Lo 2023) and to prove (Sweeney 2023). The major difference between GenAI and essay mills is that writing with GenAI is usually free, quick and easily accessible by all students, which is likely to increase the rate of student plagiarism exponentially, making it a very serious threat to academic integrity (Perkins 2023).

On the positive side, software companies have rushed to offer methods of detecting GenAI-generated writing (Rudolph et al. 2023), even though they are not yet completely reliable (Weber-Wulff et al. 2023). Detecting plagiarism with software has long been used in higher education (Foltýnek et al. 2019), and many lecturers insist on using appropriate detection tools (Meccawy et al. 2021), as research has shown they act as deterrent (Heckler et al. 2013). Unfortunately, GenAI can also be used by students to escape detection by software (Rudolph et al. 2023).

#### New definition of plagiarism under GenAI

Before the arrival of GenAI, plagiarism was defined as someone using someone else’s words or ideas and passing them off as their own to gain an advantage (Fishman 2009; Louw 2017). With the arrival of GenAI, Peters (2023) proposed a new definition of plagiarism with regards to writing for assessment: “presenting the words or ideas of another person, or those generated by an GenAI-assisted tool, without reference to the source from which the information originates, with the aim of gaining an unfair advantage in an assessment context” (translated from Peters 2023, p. 4). With this new definition, students who use GenAI to generate text, whether a sentence, a paragraph, or a complete assignment, and submit it as their own would be susceptible to sanctions. Plagiarism exists outside of the assessment context as well, but, for this article, we are examining it only within the context of an assignment.

### Higher education, traditional assessment tasks and preventing plagiarism

One of the ways to get away from the cat-and-mouse game of misusing GenAI and detecting any plagiarism is for lecturers to step away from the punishment paradigm (Rudolph et al. 2023) and to work on preventing plagiarism (Bretag 2013). Modifying assessment tasks, instructions and criteria can contribute significantly towards fostering academic integrity (Egan 2018; Sotiriadou et al. 2020). Only then will we be able to establish a culture of integrity where everyone in our institutions, lecturers and students, will value honesty, trust, respect, accountability, fairness, responsibility and courage (International Center for Academic Integrity 2021).

Despite the potential benefits of redesigning different aspects of higher-education pedagogy and assessment, there have been few changes so far (Simper et al. 2022), in either teaching or types of assignment (Williams 2014), with the result that traditional methods still prevail (Lebrun 2015). One of the reasons why there have been few modifications in the teaching and assessment practices in higher education is the lack of upskilling by university lecturers. While they have spent long years learning about their field of specialization, most lecturers have little or no training in pedagogy (Stewart 2014; Vilppu et al. 2019). Researchers explain how this lack of training has resulted in classes being taught and students evaluated in the same way in which lecturers themselves have learned while at university (Oleson and Hora 2014; Snook et al. 2021).

Exams and written coursework (essays, literature reviews, reports, annotated bibliographies, etc.) have been the traditional methods of assessment at university (Slade et al. 2022). These are usually summative and broad in thematic scope, with a tendency to concentrate on comparing individual students’ performance to established norms (Williams 2014). Typically, traditional assignments are seen as efficient and familiar, and tend to simplify the creation and grading of assessment tasks (Parker et al. 2021). They are also perceived to be objective, reliable and valid (Linden and Gonzalez 2021; Sambell et al., n.d). Of course, each discipline has different traditions, and so this article will be addressing more specifically the social sciences including business disciplines and the humanities.

When class sizes are large, lecturers are more resistant to changing the way they assess students (Bovill 2020), due to insufficient time to create or explore resources to innovate in their assessment (Oleson and Hora 2014). Large class sizes, heavy workload, accountability and student complaints force lecturers to give feedback which is more limited and aimed at justifying grades rather than promoting student learning (Winstone and Boud 2022). Too often, they will reuse the same assignments, year after year, with the unfortunate consequence of facilitating student plagiarism (Ford and Hughes 2012). A further negative repercussion from the lack of variety in assessment methods is students’ disengagement (Viegas et al. 2015), which in turn, encourages the outsourcing of assignment completion, or contract cheating (Sutherland-Smith and Dawson 2022).

## Assessment in a GenAI world: model overview

With the arrival of GenAI in education, many experts are urging lecturers to modify their assessment methods to try and curb the temptation for students to include GenAI-generated text in their assignments (Dakakni and Safa 2023; Nguyen Thanh et al. 2023; Perkins et al. 2023). When planning their courses with the view to encouraging academic integrity, lecturers would do well to adapt and use a range of assessment tasks – with varying length and weighting, with scaffolded learning, and with high relevance and authenticity for students (Lehane and Wright 2024; Miserandino 2024; Sullivan et al. 2023).

**Fig. 1:**
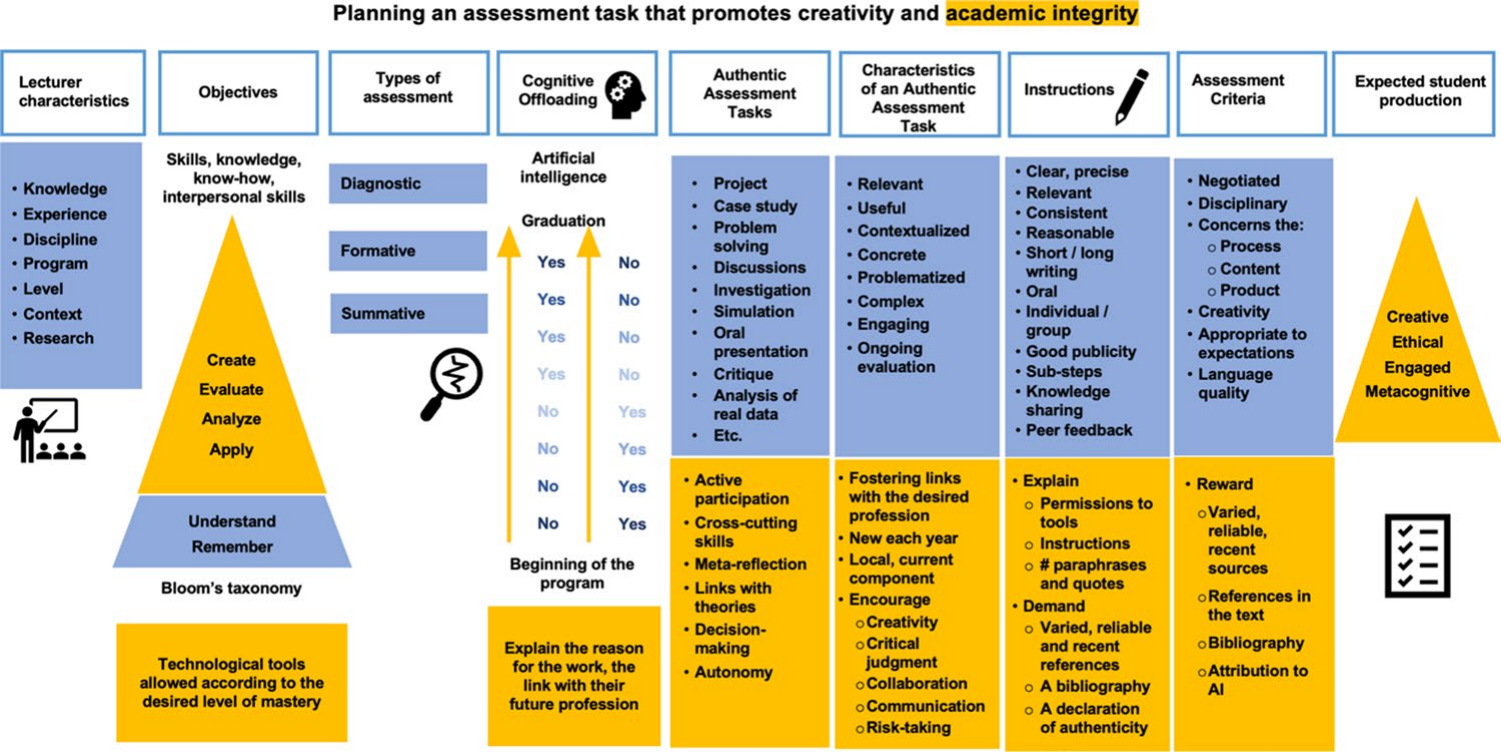
Academic Integrity and Creativity in the age of Generative Artificial Intelligence model (AICAI)

Assignment instructions and criteria need to be revised so that students know what is expected of them (Cotton et al. 2024), and how and when they can use GenAI in their assignments (Rane 2023). Clear instructions and criteria will ensure that assessment is fair, accurate, inclusive, and reflective of students’ abilities. The following model (see Fig. 1) has been elaborated to show all the elements a lecturer has to consider when planning an assessment task in the age of GenAI. The blue color represents what is essential in assessment design in general, while the gold color indicates elements that can further enhance the assignment’s compliance with the principles of academic integrity, more specifically. The model reads from left to right, but the process of assessment planning may not necessarily follow a left-to-right sequence of steps. For example, a lecturer could choose their intended type of authentic assessment, as a starting point, and then select the relevant objectives.

In the rest of this article, the AICAI model will be presented in sections, and explanations will be given to show how lecturers can promote both creativity and integrity in their students.

### Lecturer characteristics

**Fig. 2:**
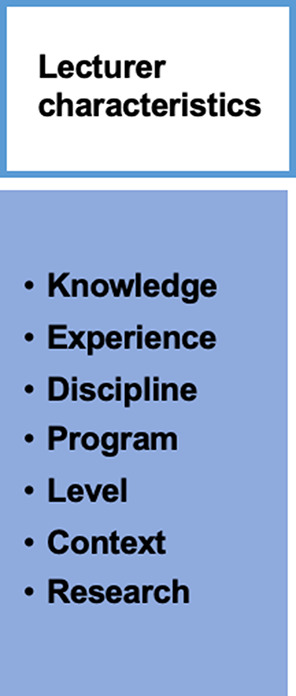
Lecturer characteristics

The first element of the AICAI model focuses on the lecturer as the agent driving the assessment process and, more specifically, on their professional characteristics and circumstances within the teaching context for which the assessment task is designed (see Fig. 2).

When planning any assessment task for a class, a lecturer must consider their own characteristics (Apostol et al. 2023), which can include: (1) the years of experience in teaching (a novice versus an experienced lecturer); (2) the number of times the course has been taught (first time or fifth); (3) the knowledge of the disciplinary content of the course; (4) the knowledge of the program in which the course is offered (whether the course is a prerequisite, for example); (5) the level of the program (undergraduate, graduate, certificate, etc.); (6) the context in which the course is offered (for example, if the geographic area or the political climate can affect the course content); and, finally, (7) whether the lecturer is doing research in the course content. As part of these seven elements, one could consider the knowledge the lecturer has of GenAI and of the existing GenAI tools in the discipline of the course taught. All these lecturer characteristics and types of knowledge can influence the choices made when planning the course assessment tasks.

### Objectives

**Fig. 3:**
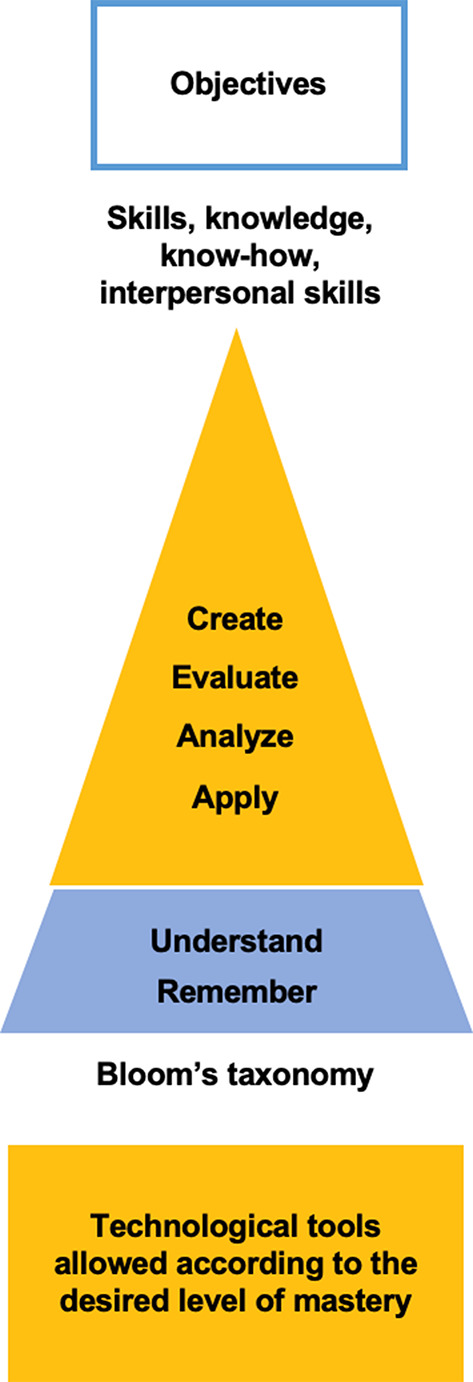
Objectives in the assessment model

Having considered their individual expertise and circumstances, a lecturer must focus on the specific objectives of the assessment task they would like to design. This is the second stage of the assessment-planning process as represented in the **AICAI** model proposed here (see Fig. 3).

When designing an assessment task, choosing objectives that are tailored to the students’ needs and interests is crucial (Sewagegn 2020). It is essential, to foster students’ engagement by explaining what they will learn, while doing this task (Fons and Torres 2021), and how it is relevant for them (Phung 2017).

As a leading concept in assessment design, Bloom’s taxonomy (Newton et al. 2020) can be used not only to determine the complexity of the task, but also to choose task objectives that will deter plagiarism. Task objectives related to the two cognitive processes positioned at the base of the pyramid – remember and understand – will result in simpler, more straightforward assignments (Adams 2015), possibly leading to higher rates of GenAI-assisted plagiarism, as remembering and understanding limit students’ interaction with the information learned (Salmons 2008). Conversely, objectives related to cognitive processes positioned towards the top of the pyramid - applying, analyzing, evaluating and creating – will engage students in tasks that are multifaceted and more challenging, therefore, harder to plagiarize using GenAI (Fons and Torres 2021). In line with the argument presented here, the Oregon State University has produced a version of Bloom’s taxonomy that shows how GenAI capabilities and distinctive human skills can be applied at all of Bloom’s levels (Oregon State University 2024). This new version of Bloom’s taxonomy can be used to choose learning objectives that will foster human skills. Assessment tasks should push students towards the higher levels of Bloom’s taxonomy. In Nursing, for example, such an assignment will require that students create a meal plan based on their analysis of a patient’s symptoms and then present it orally to classmates. They may be allowed to use GenAI tools to gather information and suggest various foods, but the final analysis would be the students’ responsibility.

#### Defining creativity

While creativity is at the top level of Bloom’s taxonomy, it is important to define it so that lecturers’ expectations of students are realistic. A standard definition of creativity involves two factors: the first factor consists of a constellation of characteristics such as novelty, originality, infrequency, or unusualness; and the second factor is related to usefulness, value, utility, effectiveness, adaptability, or appropriateness” (Acar et al. 2017, p. 133).

In a world with so much available information, it becomes difficult for students to produce something completely original, never seen before (Tanvir 2023). In fact, it is unrealistic and even counterproductive for a student, who is by definition a learner rather than an expert, to aim for such an ambitious goal (Johnson-Eilola and Selber 2007). Anderson et al. (2001, p. 85) insist that educators must define creativity when using Bloom’s taxonomy because “… many objectives in the *Create* category do not rely on originality or uniqueness”. Similarly, Johnson-Eilola and Selber (2007) explain how creativity is no longer inventing something completely new, but rather putting together an assemblage, or a patchwork of information, for a specific purpose. For students, this means finding relevant information, remixing it, adapting it to their situation and solving a given problem. And so, “if we tell students that their goal is not to create new, unique texts but to filter and remix other texts in ways that solve concrete problems or enact real social action” (Johnson-Eilola and Selber 2007, p. 380), the temptation to plagiarize or use GenAI without integrity will be strongly diminished, as the end goal will appear achievable rather than intimidating. Furthermore, using this modified definition of creativity will align better with lecturers’ pragmatic instincts to encourage creativity on a realistic and achievable scale, not necessarily originality in the “genius” sense of the word (Johnson and Clerehan 2005). Creativity that encourages students to analyze and reinterpret existing phenomena will be better suited to helping them develop critical thinking and problem-solving skills (Abe and Birabil 2022).

### Type of assessments

**Fig. 4:**
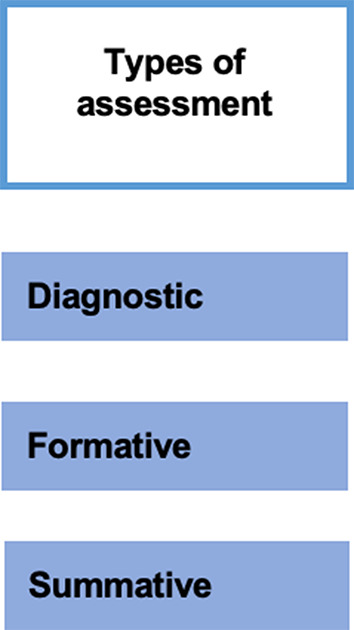
Type of assessment

The third area of consideration when developing integrity-compliant assessment in a GenAI-assisted higher-education context is the type of assignment (see Fig. 4 below).

In higher education, summative assessment tasks, when lecturers evaluate to give a grade, are the most often used type of assessment (Yan et al. 2021); they are also usually high-stakes assessments at the end of a trimester (Harrison et al. 2017). In contrast, diagnostic or formative assessment tasks, or even low-stakes assessments, are less frequently used (Yüksel and Gündüz 2017), which is unfortunate as students miss out on the potential benefits they bring. For example, at the beginning of a semester, a diagnostic writing task can inform a lecturer of the level of their students and help them calibrate the content of their course accordingly. In the context of GenAI, the same task could be used later to compare against a longer assignment and determine if students had used GenAI to plagiarize.

Formative assessment tasks can also give lecturers rich information about student learning (Trumbull and Lash 2013) and help them determine follow-up learning objectives (Leenknecht et al. 2020). However, the greatest advantage of formative assessment tasks is the feedback received by the students, giving them a chance to learn from their mistakes, discuss areas for improvement with their lecturers and, when possible, to develop further their coursework (Voinea 2018). Formative assignments are also a way to reduce plagiarism, since they alleviate the high-stakes pressure associated with summative assessment (Leung and Cheng 2017).

If formative assignments reduce the temptation to commit plagiarism, summative assessment tasks have the opposite effect. With their focus on the final product, their overall importance, fixed deadlines and limited feedback possibilities in a competitive environment, these usually higher-stakes assessments make plagiarism more tempting for students (Leung and Cheng 2017).

### Cognitive offloading

When planning an assessment task, lecturers must determine what kind of aids and assistance students will be allowed: dictionaries, notes, computers, spellcheckers, the internet, etc. Using resources or tools to help with a cognitive task is known as cognitive offloading which “has been demonstrated to improve performance across several domains (e.g., perception, memory, arithmetic, counting, and spatial reasoning)” (Risko and Gilbert 2016, p. 676). Carter (2018, p. 657) defines this process as “outsourcing” cognitive tasks to a variety of “technological gadgetry (such as iPhones, smartwatches, Google Glass and so on)” which can also be used for learning and work (Grinschgl and Neubauer 2022). In the context of GenAI, students need to understand how to use the new technology as a cognitive offloading tool, both in their studies and in their future employment, with the strategic goal of maximizing their performance (Grinschgl and Neubauer 2022).

**Fig. 5:**
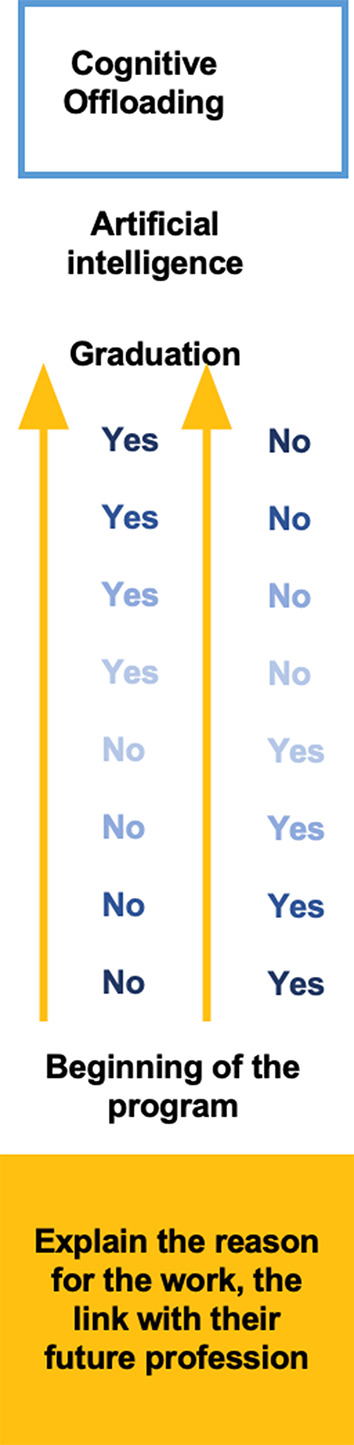
Cognitive offloading

With the arrival of GenAI in education, lecturers must also determine which GenAI tools can be used for cognitive offloading in an assessment task, when and for which purpose (see Fig. 5).

For example, in a law class, students might be allowed to use GenAI tools to help them polish the text of a brief they have written, as they are likely to be graded on their argumentative skills rather than the mechanics of writing. On the other hand, in a language class, this would not be allowed because language students are graded on their language skills, including grammar and spelling. Dawson (2020) explains that designing assessment tasks with cognitive offloading, in our case with the use of GenAI, should have three specific goals. First, the lecturer must still be able to make a judgment about the students’ abilities even if GenAI is used. Second, the assessment tasks, in which GenAI is used to reduce cognitive demands, must be designed to facilitate students reaching more comprehensive and in-dept results (Dawson 2020). Finally, the tasks should enable students to judge the outcomes of their work done with cognitive offloading.

Following these planning goals, (Dawson 2020) presents five principles for integrating cognitive offloading that lecturers must consider when they design assessment tasks:

1. “Specify learning outcomes and assessment with reference to the cognitive offloading allowed.
2. Programmatically offload lower-order outcomes once mastered.
3. Treat capability with cognitive offloading as an outcome.
4. Extend the development of evaluative judgement to include the products of cognitive offloading.
5. Use cognitive offloading authentically” (Dawson 2020, p. 46).

Dawson suggest that these principles must be considered when planning assessment tasks for a one-semester course but, even more importantly, they should be applied within a whole program, with a scaffolding or a reverse-scaffolding approach (Dawson 2020). Applying Dawson’s reverse-scaffolding approach in a business administration program would entail first-year students not being allowed to use GenAI tools to create a business plan, which is a fundamental skill to develop in the discipline. Fourth-year students, on the other hand, would be permitted access to GenAI to help them prepare their business plans, but would have to do the implementation by themselves, thereby building on the knowledge acquired by creating the plans. In other words, a lecturer designing an assessment for a course needs to consider much more than the requirements of the course itself; they need to reflect on the place of the course within the respective program before starting to look at the type of tasks which will be appropriate to offload within all the parameters already discussed.

### Authentic assessment tasks

**Fig. 6:**
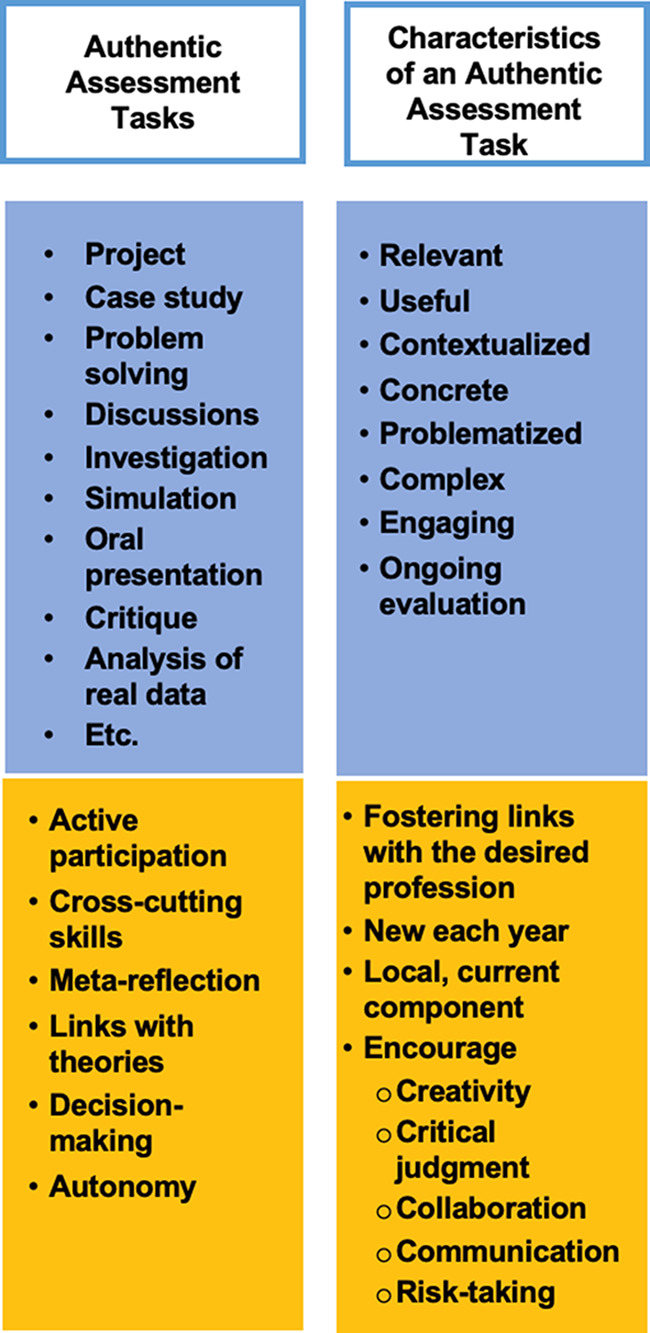
Authentic assessment tasks and their characteristics

The advent of GenAI has made it even more pressing for lecturers in higher education to adopt new types of assessment, such as authentic assessment, as a deterrent against the proliferation of plagiarism amongst students (see Fig. 6).

In the last few years, there has been an increased focus on how authentic assessment can reduce plagiarism in students’ papers (Bretag et al. 2019; Openo 2023). An authentic assessment task, according to McArthur (2023, p. 87), should shift the “focus from the real world/world of work to a richer understanding of society as a whole; secondly, to transcend a focus simply on the task, and consider instead the value of that task; and finally, […] [it should] not reinforce the status-quo but [.] instead [be] a vehicle for transformative social change”. Authentic assessment can take many forms: project, case study, problem solving, discussions, investigation, simulation, oral presentation, critique, analysis of real data. Sotiriadou et al. (2020, p. 2144) have suggested that oral assessment activities are perceived by students has “highly authentic and relevant to their employability and promote academic integrity”. Authentic oral assessment activities such as role-play or interactive oral tasks “are seen as potential types of assessment that would support the usage of GenAI as a learning tool while safeguarding the academic integrity of the assessment” (Kofinas et al. 2025, p. 6).

Authentic tasks are complex (Openo 2023) and provide a more comprehensive snapshot of students’ knowledge and skills (Hagler 2020). Researchers propose different sets of criteria to determine the level of authenticity of an assessment (Bosco and Ferns 2014; Ellis et al. 2020; Iverson et al. 2008), with the overarching criterion being that it should encourage students to engage in real-world tasks, frequently done in their chosen professions and in similar conditions (Ellis et al. 2020). Authentic tasks are thus complex problems that need to be solved (Hamdy 2015), bringing students to self-assess and reflect on their learning (Bosco and Ferns 2014; Iverson et al. 2008). Finally, these tasks should, whenever possible, involve students in a professional context outside of their courses (Bosco and Ferns 2014).

Now, with the introduction of GenAI in academia and the professional world, it’s even more important that lecturers include GenAI-assisted tools in their authentic assessment tasks. Given the presence of GenAI outside of the classroom environment, such inclusion will help students understand more easily the applicability of their assignments, and will motivate them to engage and use GenAI with integrity. Bearman and Luckin (2020) specify that assessment in general in the age of GenAI be modified (1) to maximise human endeavors; (2) to encourage students to reflect and be critical; and, finally, (3) to require of students to recognize and distinguish between their own contribution and that of the technology. In this way, students will be better prepared for the employment world, regardless of how it evolves in the future.

Although certain researchers argue that some authentic assessment tasks are likely to reduce plagiarism (Baird and Clare 2017; Bretag et al. 2019; Openo 2023), others, such as Ellis et al. (2020), have found that many students continue to commit contract cheating (a form of plagiarism) despite the authentic nature of their assessment. Thus, students’ engagement is a crucial factor, as they are the ones who “decide what is essential assessment to complete and what is not in determining their own curriculum priorities” (Sutherland-Smith and Dawson 2022, p. 93).

### Instructions

**Fig. 7:**
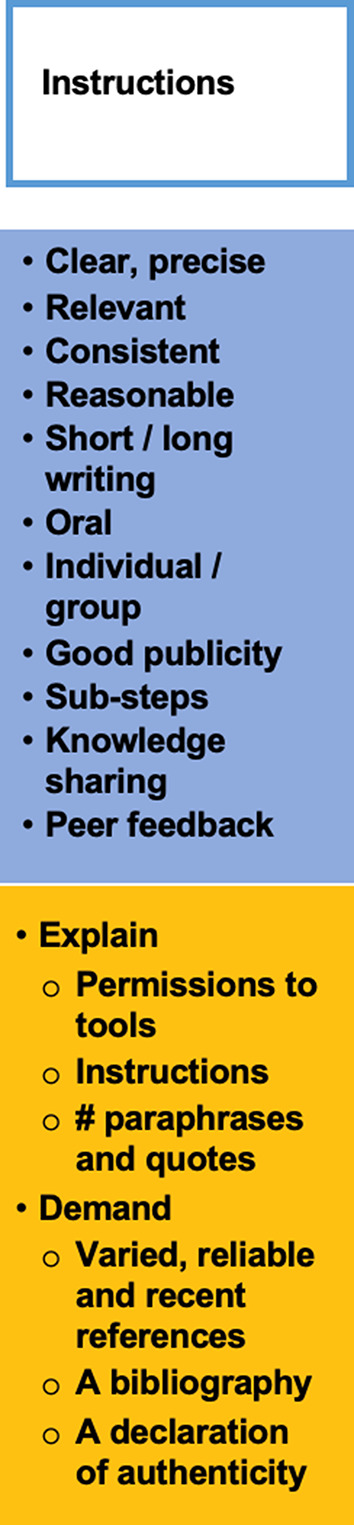
Instructions for assessment tasks

Another key element of assessment design that needs to be considered when developing GenAI-compliant assignments is the concrete instructions included in the assignment briefs (see Fig. 7).

For students to be motivated to engage in an assessment task, they need, from the beginning, to be able to understand the expectations, the purpose of the task and the lecturer’s instructions (Smith et al. 2013). Instructions are a type of contract whereby one person, the lecturer, specifies what is expected and the other person, the student, is obliged to fulfill it. It is a formal understanding and, as such, must be clear and written in a language the students can understand (Copeland et al. 2018). In the current higher-education climate, lecturers must also think of their instructions as akin to advertisements, if they are to ‘sell’ their assignments to their students. Therefore, instructions should be short, to the point, clear, precise and visually appealing. Godin (2023) builds an illuminating analogy between the genre of the instruction manual (for example, an IKEA leaflet describing how to build a library) and a lecturer’s assessment instructions, or assignment brief. The author presents many elements that lecturers could consider when writing their instructions for an assessment task, the five most relevant of which are presented here:

1. “Assume that some people *(students)* will not read the manual, no matter how clear it is or what format it takes”.
2. “If it’s important, say it a few times, in a few places”.
3. “One reason linear instructions are problematic is that the teacher doesn’t know what the student doesn’t know. So we end up including too much, just to be sure we aren’t leaving someone behind. Which is boring, so people *(students)* zone out or skip ahead”.
4. “Different people *(students)* learn in different ways, so why have only one way for them to read the manual”?
5. “If you’re not changing the instruction manual in response to user feedback, then your user manual is obsolete”.

Another set of recommendations written before the arrival of GenAI, yet still particularly relevant, are Harris’ (2015) eight instruction strategies for plagiarism prevention:

1. Be sure that the instructions are clear as poor understanding of the instructions leads to poor papers.
2. Students can choose from a list of specific topics or recent issues selected by the lecturer that must be changed every term.
3. Instructions should contain specific components designed to foster academic integrity: for example, a requirement that students need to include in their assignment’s references to a specific theory, as discussed in class. Students may also be asked to link the classroom discussion to sources identified through independent research, making it even more difficult for the assignment to be produced with the help of GenAI tools.
4. The process of producing an assignment should be spread out over a few weeks, in smaller parts, with explicit deadlines for each stage. This will help students who tend to procrastinate until the last minute to write their assignment in time, which will, in turn, reduce the temptation to plagiarize.
5. Oral presentations can complement written assignments as part of course assessment. Students who have used GenAI to write their papers, or have bought them online, will find it difficult to explain their contents facing an audience.
6. Lecturers should require the submission of annotated bibliographies with specific components in their instructions. This will encourage critical thinking on the reliability and quality of sources. For example, students could be asked to include three scientific and two professional sources in their bibliographies and explain their relevance to their assignments.
7. Instructions should require that references be recent, with the lecturer specifying a cut-off date. While this will not stop students from using GenAI to search for sources, it will oblige them to read newer materials which are less frequently mentioned on the internet and therefore less likely to have lots of other sources that refer to them.
8. Finally, Harris (2015) advises lecturers to ask students to write a “metalearning” essay on the day the assignments are handed in. These require students to reflect on what they have learned, what has proved easy or difficult in the assignment, which will make them aware of their strengths and weaknesses and will assist them with their future work. Students who have written their assignment with GenAI will have a hard time producing a credible reflection.

In summary of Harris’s eight points, it could be concluded that instructions should contain clear guidelines on what is acceptable GenAI use for each assignment. This can change from one assignment to the next, but needs to be made explicit, so that students know what the expectations are (see Spannagel (2023) for an example).

### Assessment criteria

**Fig. 8:**
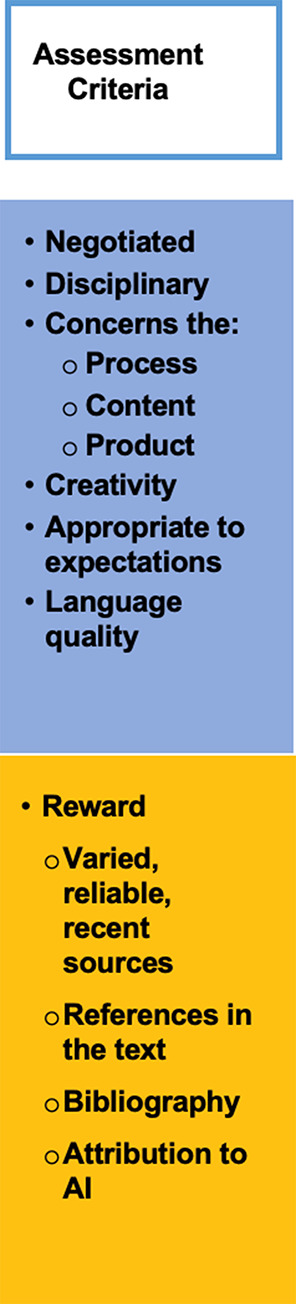
Assessment criteria

Assessment criteria (see Fig. 8 below), for example in evaluation rubrics, must also be made explicit to students before they even begin their assignments (Worth 2014), since knowing how and according to what criteria they will be graded helps students plan and complete their tasks successfully (Andrade and Du 2005; Miihkinen and Virtanen 2018).

As part of discussing assessment criteria with their students, lecturers can engage in a process of negotiation and co-creation. Students’ involvement in the selection of criteria, their weighting (in terms of percentage) and the submission deadlines will give them a voice and a sense of control, which, in turn, will make them more engaged in the task (Leslie and Gorman 2017; Meer and Chapman 2014). Discipline-specific criteria should be included to make the assignment more relevant to the students’ study goals; for example, awarding them points when their assignments refer to experiences they may encounter in their future professional lives.

Assessment criteria should cover more than just the disciplinary content of a paper. There should be criteria pertaining to the process of writing the paper (the abilities and skills students have shown to produce the assignment) as well as for the form of the final product (whether or not it respects the lecturers’ instructions) (see Table 1). The following table is the authors’ proposal for sample grading criteria that focus on content, process and product but are also aimed at promoting integrity.

**Table 1:** Criteria on content, process and product aimed at promoting integrity

Elements	Criteria	Percentage
Process	For your conclusion, write a metacognitive reflection (500 words) to explain what was easy/difficult about writing the assignment, what you learned, etc.	5%
	Explain where and how you found your sources	1%
	Highlight in yellow the places in your text where you’ve used GenAI and explain in one sentence in the footnote how you used it	2%
Content	Give the strengths and weaknesses of Smith’s theory, discussed in class, from the specific point of view presented in your work	5%
	Use authors to support the statements you include in your text	5%
	Use the statistical results of the census to support your argument	2%
Product	Give five direct quotes in your work from sources included in your bibliography	2%
	Use reference management software to produce your bibliography	2%
	Highlight five paraphrases you’ve made and put the original sentences in brackets, making sure the two sentences have the same meaning but expressed in different words	2%
	Revise your work with a GenAI tool and show the modifications	5%

No assignment can be completely “GenAI proof” but these examples of criteria focus on promoting academic integrity, making it more difficult for the students to rely solely on GenAI-produced text.

## Conclusion

The advent of GenAI in higher education has the potential to radically change teaching and learning practices and pedagogy across the world. Given the serious risks that the technology poses to the integrity of university assessments and degrees, lecturers must work to minimize the negative impact of GenAI through the conceptualization and design of assessment tasks.

**Fig. 9:**
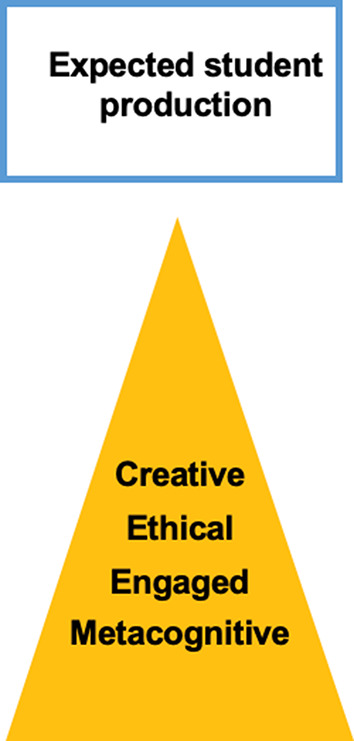
Expected student production

Notwithstanding the risks, GenAI offers unique opportunities for key stakeholders in higher education. Lecturers can use it to upgrade their assessment methods to increase creativity, integrity, engagement and student metacognition (see Fig. 9), and, consequently, to accommodate the demands of the labor market of the future. For their part, students can expect the assessment process to become fairer, better tailored to their individual and professional goals, and more rewarding, as an experience.

By combining different strands of pedagogical theory and research, the **AICAI** assessment design model has brought into focus the challenges as well as the opportunities that have emerged with the inclusion of GenAI in higher education. On a more practical level, it offers a systemic approach and advice as to how the challenges can be mitigated and benefits maximized for all parties involved in assessment.

## Data Availability

No datasets were generated or analysed during the current study.

## Footnotes

1 For this paper, the word lecturer has been used generically to include professor, instructor, sessional, and teacher.

2 For this paper, artificial intelligence tools and generative intelligence tools have been regrouped under the same GenAI concept.

## Abbreviations

GenAI: Generative Artificial Intelligence
AICAI: Academic Integrity and Creativity in the age of Generative Artificial Intelligence model
